# Genome-independent hypoxic repression of estrogen receptor alpha in breast cancer cells

**DOI:** 10.1186/s12885-017-3140-9

**Published:** 2017-03-20

**Authors:** Mercè Padró, Raymond J. Louie, Brian V. Lananna, Adam J. Krieg, Luika A. Timmerman, Denise A. Chan

**Affiliations:** 1 Department of Radiation Oncology, University of California, San Francisco, CA 94115 USA; 2 Department of Obstetrics and Gynecology, Kansas University Medical Center, Kansas City, KS 66160 USA; 3 Helen Diller Family Comprehensive Cancer Center, University of California, UCSF Mail stop 0875, 2340 Sutter Street, Room N361, San Francisco, CA 94115 USA

**Keywords:** Hypoxia, Estrogen receptor, HIF, HIF-1α, HIF-1 alpha, Endocrine therapy, Breast cancer, Tamoxifen, Aromatase inhibitor, Drug resistance, Endocrine therapy resistance

## Abstract

**Background:**

About 75–80% of breast tumors express the estrogen receptor alpha (ER-α) and are treated with endocrine-target therapeutics, making this the premier therapeutic modality in the breast cancer clinic. However, acquired resistance is common and about 20% of resistant tumors loose ER-α expression via unknown mechanisms. Inhibition of ER-α loss could improve endocrine therapeutic efficacy, benefiting a significant number of patients. Here we test whether tumor hypoxia might commonly produce ER-α loss.

**Methods:**

Using standard molecular and cellular biological assays and a work station/incubator with controllable oxygen levels, we analyze the effects of hypoxia on ER-α protein, mRNA, and transcriptional activity in a panel of independently-derived ER-α positive cell lines. These lines were chosen to represent the diverse genetic backgrounds and mutations commonly present in ER-α positive tumors. Using shRNA-mediated knockdown and overexpression studies we also elucidate the role of hypoxia-inducible factor 1-alpha (HIF-1α) in the hypoxia-induced decrease in ER-α abundance.

**Results:**

We present the first comprehensive overview of the effects of *bona fide* low environmental oxygen (hypoxia) and HIF-1α activity on ER-α abundance and transcriptional activity. We find that stabilized HIF-1α induces rapid loss of ER-α protein in all members of our diverse panel of breast cancer cell lines, which involves proteolysis rather than transcriptional repression. Reduced ER-α severely attenuates ER-α directed transcription, and inhibits cell proliferation without overt signs of cell death in the cell lines tested, despite their varying genomic backgrounds.

**Conclusions:**

These studies reveal a common hypoxia response that produces reduced ER-α expression and cell cycle stalling, and demonstrate a common role for HIF-1α in ER-α loss. We hypothesize that inhibitors of HIF-1α or the proteasome might stabilize ER-α expression in breast tumors in vivo, and work in combination with endocrine therapies to reduce resistance. Our data also suggests that disease re-occurrence in patients with ER-α positive tumors may arise from tumor cells chronically resident in hypoxic environments. We hypothesize that these non-proliferating cells may survive undetected until conditions change to oxygenate the environment, or cells eventually switch to proliferation via other signaling pathways.

**Electronic supplementary material:**

The online version of this article (doi:10.1186/s12885-017-3140-9) contains supplementary material, which is available to authorized users.

## Background

Breast tumors are classified into subtypes based on molecular and pathological characteristics that impart prognostic value or determine therapeutic treatment plans. The most common and clinically relevant feature is expression of the estrogen receptor alpha (ER-α; *ESR1*) transcription factor, which occurs in 70–80% of all breast tumors. ER-α is activated by the endogenous hormone 17-beta-estradiol (estrogen), to bind estrogen-responsive elements (ERE) in DNA and induce gene transcription (reviewed in [1, 2]). ER-α can also function as a non-DNA-binding element of other transcription complexes; and cytosolic, non-genomic functions are ascribed to specific ER-α splice variants.

Estrogen responsive genes include those required for survival and proliferation, and most ER-α -positive breast tumors are dependent on estrogen for growth. As such, several targeted therapies have been developed that either compete for estrogen binding (for example tamoxifen), or inhibit the body’s ability to produce estrogen (for example aromatase inhibitors). Most ER-α positive tumors initially respond well to these therapeutics, and ER-α expression generally imparts a better 5 year prognosis. However around 30% of ER-α positive tumors are intrinsically resistant to endocrine therapies and 30–40% of those that initially responded will become therapy resistant (reviewed in [1, 2]). Of these, about 20% will lose ER-α expression. Patients with endocrine-resistant tumors account for nearly 11,000 annual US breast cancer deaths.

### Heterogeneity of ER-α positive tumors influences therapeutic responses

As a class, ER-α positive tumors are heterogeneous in the expression of hundreds of other molecules that may influence proliferation and survival. For example, some ER-α positive tumors express activated oncogenes such as the HER2 receptor tyrosine kinase, or a mutant, activated phosphoinositide-3-kinase alpha catalytic subunit (PI3KCA; [3–5]). Other ER-α positive tumors may have inactivated tumor suppressors such as tumor protein TP53) and/or retinoblastoma (Rb). Differences may be so profound that ER-α positive tumors are found in both the luminal A and luminal B molecular subsets of breast tumors. As expected from this heterogeneity, ER-α positive tumors differ in drug responses and clinical outcomes [6, 7].

Individual ER-α positive breast tumors also exhibit significant intra-tumor heterogeneity. Tumors are considered ER-α positive by clinical histopathological standards if as few as 1% of cells stain for ER-α expression [8]. The presence of accompanying ER-α negative cells has been attributed to factors including genomic instability, epigenetic regulation, poorly-defined micro-environmental conditions, and the ongoing growth and differentiation of tumor cells from primitive, stem-cell like progenitors within the tumor mass [9]. As the use of specifically-targeted therapeutics increases, intra-tumor heterogeneity becomes an increasingly important factor in treatment efficacy [10]. The widespread use of hormone targeted therapeutics and prevalence of resistance, makes understanding the intra- and inter-tumor heterogeneity of ER-α positive tumor responses of paramount clinical importance. Thus, experimental analysis of only one or a few ER-α positive cell lines in preclinical studies cannot accurately reveal the diversity of potential responses among this class of tumors, nor predict the prevalence of various therapeutic responses.

### HIF-1α is a key regulator of adaptation to hypoxic conditions

Conditions of low oxygen (i.e. hypoxia) are hallmarks of solid tumors, and cells undergo profound alterations to survive this environment (reviewed in [11]). Hypoxia promotes the growth of tumors that have reduced apoptotic abilities [12] increased metastatic potential [13] and increased genomic instability [14–16]. Hypoxia is a poor-prognosis factor for breast cancer in terms of tumor recurrence, tumor aggressiveness, disease-free survival and overall survival [17, 18]. Hypoxic conditions occur in poorly vascularized regions of rapidly growing tumors, areas located between 70 and 100 μm from blood vessels [19]. Highly transformed breast cancer cells orchestrate a varety of complex responses to survive hypoxic conditions, allowing, for example, uninterrupted protein synthesis via constitutively activated mechanistic target of rapamycin (mTOR) signaling [20]. In contrast, non-transformed breast derived cell lines respond to hypoxia (1% O_2_) by rapid inhibition of protein synthesis (reviewed in [21]).

Many features of hypoxic adaptation rely on activation of the hypoxia inducible factor (HIF) family of transcription factors. HIF functions as a heterodimer composed of an α-subunit (HIF-1α, HIF-2α or HIF-3α) and a β-subunit (HIF-1β). HIF-1α, and HIF-2α are the main transcription factors involved in oxygen level sensing and cell response (reviewed in [11, 16]) while HIF-1β (ARNT) is constitutively expressed in the nucleus independent of oxygen levels. Under normoxic conditions HIF-1α is hydroxylated on proline 402 and proline 564 by a family of prolyl-4-hydroxylases (PHDs;[22, 23]). HIF-1α hydroxylation allows recognition by the von Hippel-Lindau (VHL) tumor suppressor, which targets HIF-1α to the proteasome for degradation. Under hypoxic conditions HIF-1α degradation is impaired, allowing nuclear translocation and hetero-dimerization with HIF-1β on hypoxic-response elements (HRE). HIF-directed transcription induces genes involved in angiogenesis, glycosylation, tissue remodeling, metabolism, and cell proliferation [24–26]. HIF-1α is an independent predictor of poor response to chemo-endocrine therapy, and is negatively associated with disease-free survival in ER-α positive but not ER-α negative patients [27].

Conflicting observations regarding hypoxic alterations in *ESR1* transcription, ER-α protein stability, and ER-α transcriptional activity appear in the literature [28–31]. Some of these reports use cell culture with low environmental oxygen, but more often they use treatment with chemicals that can stabilize HIF-1α to the mimic hypoxic environment. With regard to breast cancer, these studies also primarily rely on the singular cell line MCF7, or a cell line of unknown lineage and genomic content, ZR-75. While this literature generally suggests that hypoxia promotes decreased ER-α expression, it does not present a coherent mechanistic picture that could guide improvements in hormonal therapeutics.

### ER-α protein levels are reduced in hypoxic environments

In the current study, we analyze the effect of hypoxia and HIF expression on ER-α using a panel of ten independently-derived ER-α positive cell lines. This panel has been previously characterized by comparative genomic hybridization, mRNA expression profiling, total genome sequencing, various high throughput analyses of protein expression and activation status, and comprehensive drug panel responses (for example, see [7, 32, 33]). Combined, these cell lines capture a significant amount of the diverse genetic backgrounds and accompanying mutations commonly present in ER-α positive tumors. Cell lines in our study such as BT474 and MDA-MB-361 co-express and constitutively activate the HER2 receptor tyrosine kinase; five of these cell lines bear activating mutations in the phosphatidylinositol-4, 5-bisphosphate 3-kinase subunit PIKC3A; five have mutated TP53 genes, and lines such as HCC1428 and MDA-MB-175 maintain wildtype alleles of all of these genes (Additional file 1; [3–5, 33]). Despite this genomic and drug response diversity we find that hypoxia commonly functions through HIF-1α to reduce ER-α protein levels, impede ER-α directed transcription, and inhibit estrogen-dependent cell proliferation. The latter occurs even in cells that co-express receptor tyrosine kinases such as HER2, which are known to drive breast tumor proliferation in other settings.

## Methods

### Cell culture

LY2, MCF7, CAMA-1, MDA-MB-175, MDA-MB-361, MDA-MB-231 and MDA-MB-435 cell lines were cultured in DMEM. BT474, T47D, ZR75B, MPE600, HCC1428 cell lines were cultured in RPMI. All cell lines were obtained as a kind gift from Dr. Joe Gray, (Oregon Health Sciences University, USA), and maintained with 10% Fetal Bovine Serum (FBS) at 37 °C in 5% CO_2_ and 21% O_2_. All cell lines were verified by short tandem repeat (STR) genotyping. Genomic DNA was extracted by Wizard SV Genomic DNA purification system (Promega). STR profiles were compared with publically available profiles using Promega Powerplex 1.2.

### Reagents

**Antibodies** used in this report are as follows: ER-α (clone HC-20, Santa Cruz), HIF-1α (BD Bioscience), HIF-2α (NB100-132, Novus Biologicals), phospho-p70 S6 kinase (p-p70-S6K; 9205, Cell Signaling Technology), phospho-4E-BP1 (2855, Cell Signaling Technology), β-Actin (clone AC-15, Sigma-Aldrich), Alexa Fluor- 488 (A-11008, Invitrogen), Alexa Fluor-594 (A11012, Invitrogen), HRP-anti-Mouse IgG (NA931V, GE Healthcare), and HRP-anti-Rabbit IgG (NA934V, GE Healthcare). **Reagents** used in this study are estrogen (17-β-estradiol) used at 10 nM (Sigma-Aldrich), MG132 used at 10 uM (Cayman Chemical).

### Western blot

Western blots were performed as previously [34]. Briefly, cells were lysed with urea lysis buffer (9 M urea, 150 mM, β-mercaptoethanol and 75 mM Tris pH 7.4), or RIPA buffer (Cell Signaling #9806), sonicated for 30 s and centrifuge at 15,00 rpm at 4C for 30 min. Protein quantification was performed by Bradford (BioRad Cat.500-0205), and 20–100 μg of protein were loaded in each well of a polyacrylamide gel. PVDF membranes were blocked with 5% milk in TBS-T/0.05% (Tris Buffered Saline with Tween 20 to 0.05%) at room temperature (RT) for 30 min, primary antibodies were incubated overnight at 4C in TBS-T/0.05%. After washing with TBS-T/0.05%, secondary incubation was performed at RT for 45 min followed by TBST/0.05% washes. Western blot signal was detected using Enhanced Chemiluminescent (ECL) substrate (Pierce 32106, or GE Healthcare RPN2235) in a FlourChemE machine. Exposures were chosen to provide maximum visual information about the changes in band intensity without causing overexposures that would obscure faint signals in neighboring lanes. Each western blot was repeated from 3 to 6 times, and the averages and standard deviations for the intensities of the western blot replicates for each figure are graphed ﻿and presented in tabular form in the Additional files, as indicated throughout the manuscript.

### Immune fluorescence

Cells were cultured on glass coverslips in six well plates, using complete media (RPMI or DMEM) with phenol red, supplemented with 10% FBS. Hypoxic samples were placed into the HypOxygen H35 Workstation for 48 h. Coverslips were washed twice in Phosphate Buffered Saline (PBS), fixed in Acetone 10 min at -20C, PBS washed, and nonspecific antibody binding blocked with 10% Bovine Serum Albumen (BSA) and 5% goat serum. Antibodies specific for the estrogen receptor alpha chain (Santa Cruz Biotechnology, HC-20 sc-543) were used at 1:100, anti- rabbit-488 (Molecular Probes/Invitrogen) at 1:1000. Nuclei were counterstained with DAPI (4',6-diamidino-2-phenylindole).

### Plasmids

Plasmid transfection was performed using Lipofectamine (Invitrogen) and Plus Reagent (Invitrogen) in Opti-MEM. ShScramble, sh*HIF1A* and sh*HIF2A* plasmids were previously described [34], HIF-1αODD was used to produce stabilize HIF-1α at normoxia [23] pCMV-hER-α was used to overexpress ER-α [35]. Reporter assays used ALT-4, a plasmid encoding canonical Estrogen Response DNA binding sequences (Estrogen Response Elements, ERE) controlling expression of firefly luciferase [35].

### Hypoxia treatment

Cells were subjected to 1% O_2_ for the specified time (HypOxygen H35 Workstation). Cells were passaged under normoxic conditions but cultured and harvested inside the hypoxia chamber.

### ER-α reporter assays and analysis of media estrogenic effects

To test the estrogenic effects of our normal culture media, cells were transfected with a plasmid encoding canonical Estrogen Response DNA binding sequences (Estrogen Response Elements, ERE) controlling expression of firefly luciferase [35]. The media we tested included: 1) “standard growth media” containing phenol red, (DMEM for MCF7 or RPMI for BT474, T47D and ZR75B) with 10% FBS; or 2) “E_2_ (−), estradiol-free” composed of phenol red-free DMEM with 10% charcoal stripped FBS; or 3) “E_2_ (+), defined estrogen media”, composed of phenol red-free DMEM, 10% charcoal stripped FBS supplemented with 10 nM estradiol. Cells incubated with either standard growth media or cells grown in defined estrogen medium had similarly high levels of luciferase signal (Additional file 2). Since there was no difference between the standard media and the estrogen-defined, phenol red-free media, all experiments, ER-α transcriptional activity assays and proliferation assays were performed in standard media.

For experiments in Fig. 4, cells were transfected with a plasmid encoding canonical Estrogen Response DNA binding sequences (Estrogen Response Elements, ERE) controlling expression of firefly luciferase [35] and incubated at normoxia or at hypoxia for 48 h.

All luciferase activity was determined by Dual-Glo Luciferase assay reagent (Promega) measured in a Monolight 2010 Luminometer (Promega). Firefly luciferase was normalized to protein concentration.

### In vitro growth curves

One hundred thousand cells were plated on 6-well plates in triplicate. The following day, cells were washed with PBS, and phenol red free DMEM with 10% charcoal stripped FBS was added. 10 nM estradiol was added for E_2_ (+) media or ethanol carrier for the E_2_ (−) media. Every 3 to 4 days, cells were counted.

### Cell cycle analysis

Cells were plated into 6-well plates and cultured under appropriate conditions for 3 days (MCF7, T47D, ZR-75-B) or 6 days (BT474). Cells were harvested, fixed and permeabilized in suspension in 70% ethanol, nuclei were stained with 40 μg/ml propidium iodide and 100 μg/ml RNAse A. DNA content was analyzed using an Accuri C6 Fluorescence Activated Cell Sorter (BD Biosciences). At least 150,000 cells were analyzed per sample. Cell cycle fractions were determined using the cell cycle analysis component in the FLOWJO software package (FLOWJO Enterprise).

### Real-time quantitative reverse transcriptase PCR

Total RNA was extracted from cells using RNeasy Mini Kit (Qiagen) following the manufacturer’s instructions. cDNA was generated from 1.5 μg of RNA using iScript (BioRad) following the manufacturer’s instruction. Power SYBR Green PCR reactions were performed in triplicate for each sample and analyzed using the AB Step One Plus sequence detection system. Data were normalized to TBP levels.

### Statistical analysis

Student’s *t*-test was used to determine significance. All error bars represent the standard error of the mean. Two way ANOVA was performed for the growth curve experiments in Fig. 5 using the Graphpad Software package. Student’s *t*-test was also used to analyze whether our data would reveal enhanced proteolysis of the estrogen receptor alpha under hypoxic conditions reported in Fig. 3d and Additional file 3D . Although we may not have thoroughly inhibited proteolysis in these experiments, the MCF7 sample showed a statistically clear increase in protein stability with MG132 treatment in hypoxic conditions versus normoxic conditions, (significance level (alpha) = 0.05, *p* = 0.044), Additional file 3D. Data for the other three cell lines did not reach this significance, with p values for BT474 of 0.765, for T47D of 0.98, and for ZR-75-B of 0.69.

## Results

### Hypoxia reduces ER-α protein levels

**Fig. 1:**
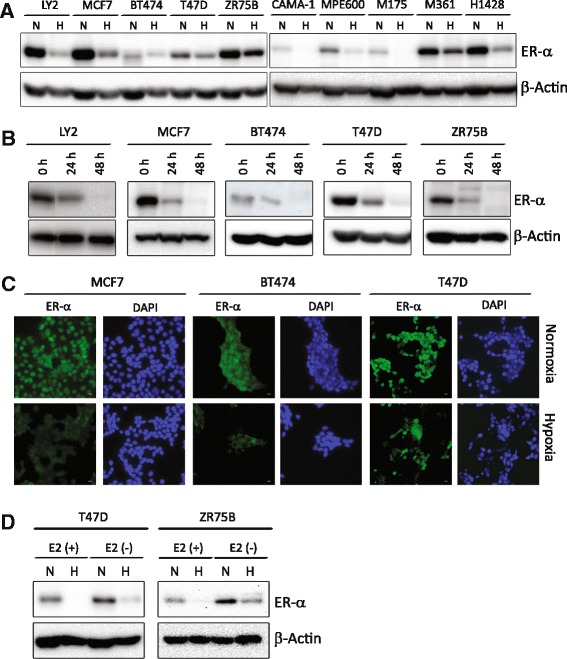
Hypoxia decreases estrogen receptor-α protein. **a** Representative western blot of ER-α protein from LY2, MCF7, BT474, T47D, ZR75B, CAMA-1, MPE600, MDA-MB-175, MDA-MB-361 and HCC1428 cells grown at normoxia (N) or treated with hypoxia (H)(1% O_2_, 24 h). **b** Representative western blot of ER-α protein from LY2, MCF7, BT474, T47D, and ZR75B at 24 or 48 h of hypoxia (1% O_2_) or normoxia (0 h). β-actin is used as a loading control. **c** Immune fluorescence of ER-α (*green*), DAPI (*blue*) nuclei, scale bar measures 25 μm. **d** Western blot of ER-α protein in ZR75B and T47D cells grown at normoxia (N) or treated with hypoxia (H) (1% O_2_, 24 h). E_2_ (+) is phenol red-free DMEM with 10% charcoal stripped FBS supplemented with 10 nM estradiol and E_2_ (−) is estrogen-free media (phenol red-free DMEM with 10% charcoal stripped FBS)

To more systematically determine the contribution of hypoxia to ER-α expression and function, we exposed a diverse set of ER-α positive cell lines to controlled, low levels of environmental oxygen (1% O_2_). The cell lines are derived from ten independent ER-α positive primary tumors, providing a diverse representation of tumor genomes found in ER-α positive breast cancer patients (Additional file 1); [33]. Protein levels of ER-α were determined by Western blot after culture at either normoxia or hypoxia (1% O_2_) for 24 h. All ten tumor cell lines exhibited a significant reduction in ER-α under this acute hypoxic condition (Fig. 1a; Additional file 4A; Additional file 5). To evaluate whether ER-α repression is transient or persistent, longer hypoxic treatment (48 h) was analyzed in five of these cell lines (LY2, MCF7, BT474, T47D and ZR75B). We found that ER-α protein levels continued to decrease over time in hypoxia in each cell line (Fig. 1b; Additional file 4B; Additional file 6). Thus longer hypoxic exposure causes stronger repression of ER-α, suggesting an important role of hypoxia in ER-α regulation. In immune-fluorescence studies, we tested whether the decrease in ER-α protein occurred in the entire population of cells or just in a few strongly expressing individual cells. We assessed ER-α expression after 48 h of culture at 1% oxygen in MCF7, BT474 and T47D. As expected from the western blot data, the protein level decrease differed for each cell line. However in each cell line tested, a strong reduction in ER-α expression was observed in the entire population of cells (Fig. 1c, green). Thus, the hypoxia-dependent decrease in the level of ER-α occurs in all cells in a population regardless of basal expression levels.

Estrogen or estrogenic surrogates such a phenol red (a common constituent of cell culture media) or various constituents of FBS, have been shown to simultaneously activate and induce degradation of ER-α (for example: [36] reviewed in [37]). Since our standard culture media contains phenol red and FBS and could therefore be considered to be constitutively activating ER-α, we tested whether the level of unoccupied ER-α would also be decreased under hypoxic conditions. We found that culture in the presence of charcoal-stripped serum and phenol red free media without added estradiol dramatically increased ER-α levels under normoxia in two representative cell lines in our collection (Fig. 1d, E2(−) N versus E2 (+) N lanes; Additional file 4C). However hypoxia still substantially reduced those ER-α levels in the absence of estrogen (E2(−) H versus (E2(−) N lanes).

### HIF-1α activity reduces expression of ER-α

**Fig. 2:**
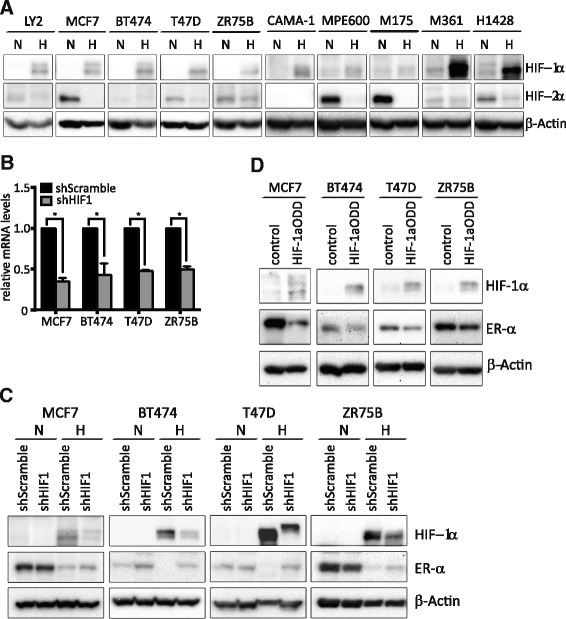
Hypoxic repression of ER-α is dependent on HIF-1α. **a** Representative western blots of HIF-1α, HIF-2α and β-actin protein in ten ER-positive cell lines grown at normoxia or hypoxia (1% O_2_, 24 h). **b** qPCR analysis of *HIF1A* mRNA levels in MCF7, BT474, T47D and ZR75B transfected with shScramble or sh*HIF1A*. Relative *HIF1A* mRNA levels normalized to *TBP*. (Change in *HIF1A* levels: *p < 0.0001 MCF7, *p = 0.012 BT474, *p < 0.0001 T47D, *p = 0.0046 ZR75B). **c** Representative western blots of HIF-1α, ER-α and β-actin protein from MCF7, BT474, T47D and ZR75B with either shScramble or sh*HIF1A* at normoxia and hypoxia (1% O_2_, 24 h). β-actin is used as a loading control. **d** Representative western blot of HIF-1α, ER-α and β-actin protein from MCF7, BT474, T47D and ZR75B with or without the transfection of stabilized HIF-1α (HIF-1αODD)

In view of the predominant role of the HIF family of transcription factors in the regulation of the hypoxic response, we next investigated whether HIF activity regulates ER-α protein levels, and whether a particular inducible HIF family member could be implicated in the breast. By western blot, we found that all cell lines in our panel exhibit a clear induction of HIF-1α under hypoxic conditions (Fig. 2a; Additional file 7C; Additional file 8). In contrast, we found that HIF-2α protein levels were not reliably induced, or in some cases were decreased by hypoxia (Fig. 2a; lanes N (normoxia) versus H (hypoxia)). These data suggested that if there was a uniform mechanism which decreased ER-α protein levels in hypoxia, it was more likely to function through HIF-1α. This conclusion is consistent with reports that HIF-1α is critical for the metastatic progression of breast cancer [38] while HIF-2α is expressed in a tissue restricted fashion that does not include the mammary gland (reviewed in [11]).

To directly investigate the role of HIF-1α in ER-α regulation, *HIF1A* was silenced by stably expressed shRNAs [34] in four genetically diverse ER-positive cell lines: MCF7, BT474, T47D and ZR75B. Knockdown was confirmed by qPCR (Fig. 2b, mRNA levels), and HIF-1α protein levels were determined by western blot (Fig. 2c; Additional file 7D; Additional file 9). *HIF1A* mRNA levels were reduced by about 50% in all cell lines. Silencing *HIF1A* in these lines partially prevented the hypoxic reduction in ER-α, implicating HIF-1α as a common mediator of ER-α modulation (Fig. 2c, H (hypoxic culture) lanes, shScramble versus sh*HIF1A*). In contrast, *HIF1A* knockdown did not affect ER-α protein levels in normoxic conditions, since HIF-1α is not stable under normoxia (Fig. 2c, N (normoxic culture), lanes shScramble versus sh*HIF1A*). In contrast, similar *HIF2A* knockdown experiments did not increase ER-α protein levels in either hypoxic or normoxic conditions (Additional file 7A, *HIF2A * mRNA; 7B, ER alpha protein; 7F western blot quantitation; Additional file 10). Finally, we tested whether overexpression of an oxygen-insensitive, stable HIF-1α allele (HIF-1αODD, [22]) would decrease ER-α levels in normoxic conditions, thereby isolating potential other effects of hypoxia from effects of HIF-1α. In transient transfection studies we found that stable HIF-1α can reduce ER-α protein levels (Fig. 2d; Additional file 7E; Additional file 11). Taken together, these findings indicate that HIF-1α reduces ER-α levels in response to hypoxia in breast cancer. Our results demonstrate that this is a general phenomenon, occurring in the context of different breast tumor genomes.

### ER-α is post-transcriptionally regulated by hypoxia

**Fig. 3:**
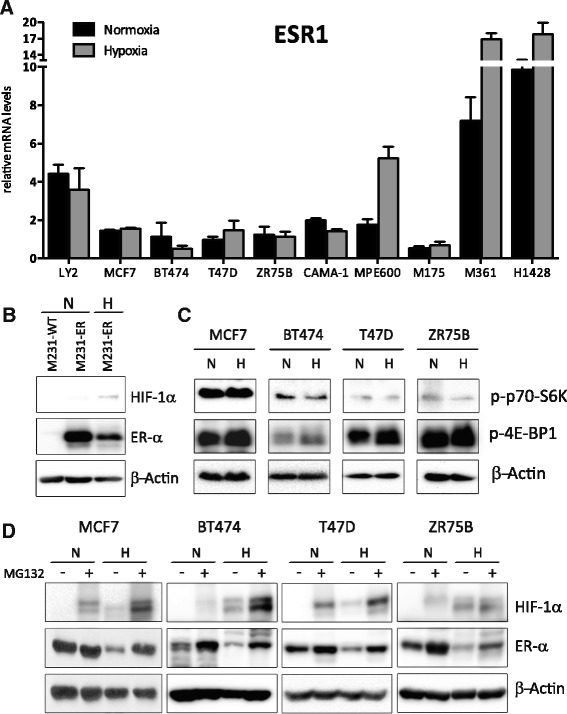
ER-α is post-transcriptionally regulated by hypoxia. **a** *ESR1* mRNA levels were compared between normoxic conditions (*black columns*) and hypoxia treatment (1% O_2_, 24 h) (*gray columns*) in a panel of ten ER-positive cell lines (Change in *ESR1* mRNA levels: *p* = 0.56 LY2, *p* = 0.24 MCF7, *p* = 0.48 BT474, *p* = 0.31 T47D, *p* = 0.82 ZR75B, *p* = 0.07 CAMA-1, *p = 0.04 MPE600, *p* = 0.55 MDA-MB-175, *p = 0.03 MDA-MB-361, *p* = 0.18 HCC1428). Levels were normalized to *TBP*. Data are represented as mean ± SEM (from duplicate experiments). **b** Representative western blot of MDA-MB-231 wild-type cells at normoxia or MDA-MB-231 overexpressing ER-α at normoxia and hypoxia (1% O_2_, 24 h) for HIF-1α, ER-α and β-actin. **c** Representative western blot of phospho-p70-S6K, phospho-4EBP1 and β-actin protein from MCF7, BT474, T47D, ZR75B at normoxia or hypoxia (1% O_2_, 24 h). **d** Representative western blot of MCF7, BT474, T47D and ZR75B at normoxic or hypoxic conditions (1% O_2_, 16 h) untreated or treated with MG132 (10 mM, 16 h) for HIF-1α, ER-α and β-actin

We next investigated whether the decreased ER-α protein levels observed under hypoxic conditions were a consequence of reduced *ESR1* mRNA abundance, as seen by some investigators [29, 30] but not by others [31]. To this end, we compared *ESR1* mRNA levels in our panel of ten ER-positive tumor cell lines following normoxic or hypoxic treatment (1% O_2_, 24 h). We found that mRNA levels of *ESR1* were essentially unaffected in eight of the ten cell lines (Fig. 3a). In the other two lines, MPE600 and MDA-MB-361, *ESR1* message levels increased 2–3 fold. However, all 10 of these cell lines exhibited decreased ER-α protein levels in response to hypoxia. These results indicate that *ESR1* is not transcriptionally regulated by HIF-1α activity in any of the different genetic backgrounds represented by the cells lines use in our study.

ER-α levels might be decreased under hypoxic conditions if protein synthesis were inhibited, a phenomena which is reported to occur under anoxic conditions [39–41] and in non-transformed cells or weakly transformed cells under a variety of less severe O_2_ tensions [20]. While this has not been attributed to HIF-1α activity, we tested whether protein synthesis was inhibited in our hypoxic cultures (1% O_2_), by testing for activating phosphorylation of two canonical regulators of protein synthesis: 4E-BP1 (a regulator of cap-dependent translation initiation), and p70 S6 Kinase (a regulator of polypeptide initiation and chain elongation). We found no decrease in activating phosphorylation of either of these proteins in hypoxic versus normoxic cultures (Fig. 3c N versus H; Additional file 3C; Additional file 12), indicating that global protein synthesis is not inhibited in our hypoxic cultures. In support of these findings, other reports indicate that in contrast to non-transformed cells, highly transformed breast tumor cell lines such as MCF7 and BT474 continue mRNA translation under hypoxic conditions similar to ours (1% O_2_ [20]. Thus, we conclude that decreased translation of *ESR1* mRNA is not the major cause of the reduced ER-α protein levels we observed.

We hypothesized that ER-α might be post-transcriptionally regulated. To test this, we treated four diverse ER-α -positive breast cancer cell lines (MCF7, BT474, T47D, and ZR75B) with the proteasome inhibitor MG132 (10 μM, 16 h). In each cell line, culture under hypoxic conditions with MG132 increased ER-α protein levels, despite their varying genetic backgrounds (Fig. 3d; Additional file 3D). We tested whether our experiments would reveal that this represented an increase in proteolysis under hypoxic versus normoxic conditions. Although we may not have inhibited proteolysis completely, we found that indeed MCF7 shows a statistically significant increase in ER-α protein levels by MG132 treatment in hypoxic conditions (alpha = 0.05, *p* = 0.044), Additional file 3D, Additional file 13. Data for the other three cell lines does not reach this significance, perhaps due to incomplete proteolysis inhibition.

We conclude that proteolysis plays a large role in the hypoxic regulation of ER-α. Analysis of mRNA levels in these experiments confirmed that increased levels of ER-α were not due to increased transcription of *ESR1* with MG132 treatment (Additional file 3A). HIF-1α levels were also determined as a positive control for MG132 proteasome inhibitory activity (Fig. 3d; Additional file 3D; Additional file 14).

We further confirmed the posttranslational regulation of ER-α by overexpressing ER-α under the control of a strong hypoxia-insensitive promoter (cytomegalovirus; CMV; [35]) in the ER-negative cell line MDA-MB-231. We found that hypoxia (1% O_2_, 24 h) potently reduced ER-α protein levels in this line (Fig. 3b; Additional file 3B; Additional file 15). Furthermore, this experiment uniquely revealed that the mechanisms regulating ER-α protein abundance during hypoxia may not be limited to luminal, ER-α positive subtypes of breast cancer, but may be more widely activated by hypoxia in very disparate subtypes of breast tumors such as the claudin low, ER-α negative subtype represented by MDA-MB-231. We note that the increase in ER-α with MG132 treatment occurred although levels of HIF-1α were also increased. Taken together, these data suggest that proteasome activity is epistatic to HIF-1α in decreasing ER-α protein levels during hypoxia.

### Hypoxia represses ER-α directed transcription

**Fig. 4:**
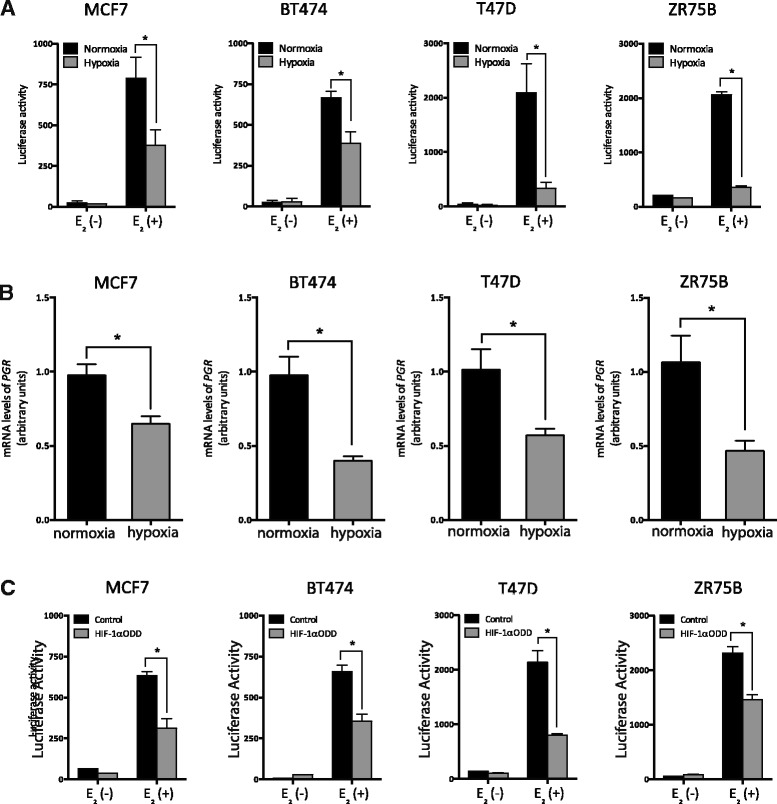
Hypoxia represses ER activity. E_2_ (−) is estrogen-free media (phenol red-free DMEM with 10% charcoal stripped FBS). E_2_ (+) is phenol red-free DMEM with 10% charcoal stripped FBS supplemented with 10 nM estradiol. **a** ER transcriptional activity analyzed at normoxia or at 48 h of 1% O_2_ using an ERE-directed luciferase reporter construct [35]. (MCF7: *p = 0.043, BT474: *p = 0.004, T47D: *p = 0.032, ZR75B: *p = 0.001). **b** qPCR analysis of mRNA levels for endogenous *PRG* mRNA transcripts at normoxic or hypoxic conditions (1% O_2_, 24 h). Relative mRNA levels normalized to *TBP*. (MCF7: *p = 0.049, BT474: *p = 0.014, T47D: *p = 0.032, ZR75B: *p = 0.047). **c** ER activity analyzed in control cells or cells transfected with a stabilized HIF-1α allele (HIF-1αODD) using an ERE-directed luciferase reporter construct [35]. (MCF7: *p = 0.036, BT474: *p = 0.034, T47D: *p < 0.001, ZR75B: *p = 0.026)

We hypothesized that reduced levels of ER-α protein would result in a concomitant decrease in ER-α-directed transcription. However other reports suggest that hypoxia can induce ligand-independent transcriptional activation of ER-α [42] or enhance ER-α ligand-dependent transcription [43]. To rigorously analyze changes in ER-α activity, we first validated a standard reporter assay and defined media conditions using cells transfected with a plasmid encoding firefly luciferase under transcriptional control of an Estrogen Response Element (ERE) -regulated promoter (Additional file 2; [35]). We then optimized growth conditions to measure ligand-dependent induction of the ERE reporter, using phenol-red free media supplemented with charcoal stripped serum to remove constitutive estrogenic influences from our cultures. In MCF7, BT474, T47D and ZR75B transfection experiments we found that the reporter was correctly silent in the absence of added estradiol, and correctly induced with the addition of physiological levels of estradiol under normoxic conditions (Fig. 4a. Black bars). Culture with estradiol under hypoxic conditions (1% O_2_, 48 h) revealed a strong and significant reduction of ER-α activity relative to normoxic culture conditions (approximately 2–6 fold; Fig. 4a E_2_(+), black versus gray bars) in each cell line tested. Similarly, transcription of the progesterone receptor (*PGR*), a canonical estrogen receptor target gene was similarly reduced (Fig. 4b). Finally, we tested the influence of HIF-1α on ER-α transcriptional activity by transient overexpression of a stabilized HIF-1α variant (HIF-1αODD) under normoxic conditions, which also attenuated reporter gene activity in response to estradiol stimulation (Fig. 4c, E_2_(+), grey versus black bars). Taken together, our results indicate that hypoxic induction of HIF-1α commonly decreases the levels of ER-α protein, which in turn reduce ER-α transcriptional activity in a variety of common breast tumor genetic backgrounds.

### Hypoxia-induced decrease in of ER-α activity inhibits proliferation

**Fig. 5:**
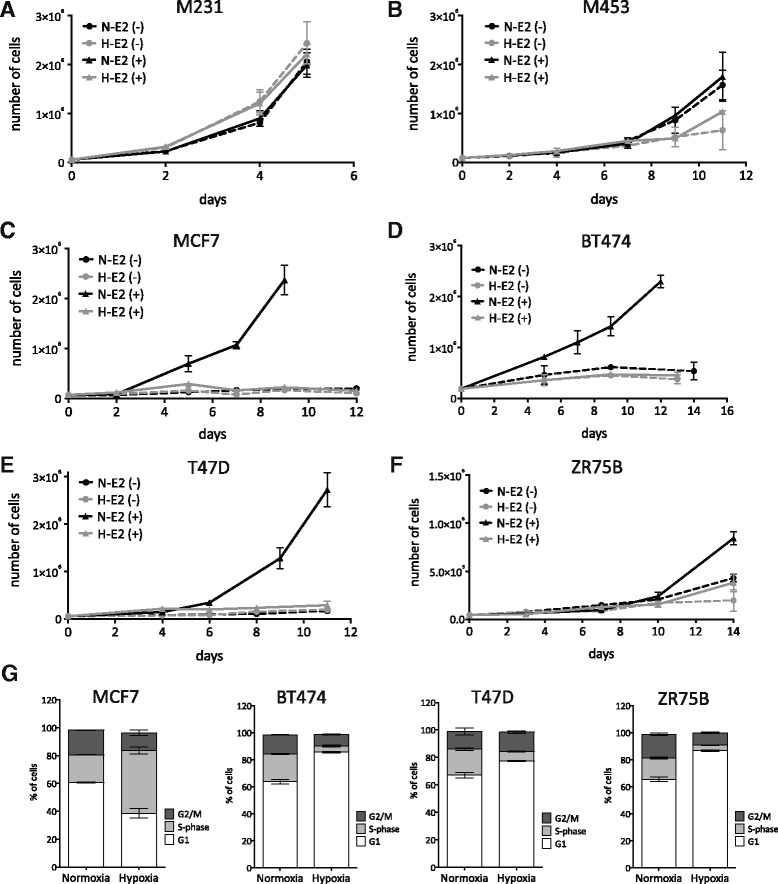
Hypoxic repression of ER activity inhibits proliferation. In vitro growth curves of cells grown with or without estradiol (E_2_) and at normoxia or hypoxia (1% O_2_). **a** MDA-MB-231, **b** MDA-MB-453, **c** MCF7, **d** BT474, **e** T47D and **f** ZR75B. *Dashed lines* are estrogen-free media, whereas *solid lines* contain 10 nM of estradiol. *Black lines* represent normoxic conditions, whereas *gray lines* are cells under hypoxic conditions. Two-way ANOVA analysis: **a** *p* > 0.05 MDA-MB-231, **b** *p* > 0.05 MDA-MB-453, **c** *p < 0.001 MCF7, **d** *p < 0.001 BT474, **e** *p < 0.001 T47D, **f** *p < 0.001 ZR75B. **g** Comparison of cell cycle distributions for cultures grown for 3 days at normoxia or hypoxia (1% O_2_). (T-student test *p < 0.001 MCF7, *p < 0.001 BT474, *p = 0.001 T47D, *p < 0.001 ZR75B). BT474 analysis was at a 6 day time point

Tumor cells might compensate for loss of ER-α activity under hypoxic conditions by switching to reliance on an alternate signaling pathway that could allow continued proliferation. For example, HER2^+^/ ER-α positive tumors represented by cell lines such as BT474 might simply switch to reliance on HER2 activity in hypoxic environments. To test the extent to which proliferation was impaired by a decrease in ER-α in hypoxic conditions, we first tested whether our ER-α positive breast cancer cell lines were dependent on estrogen for growth under normoxic conditions. As demonstrated by growth curves assays, estradiol addition to phenol red-free media containing charcoal stripped serum was required for substantial growth (Fig. 5c-f, N-E2(+) versus N-E2(−), black solid versus black dashed lines). This was not true for exemplar ER-α negative breast cancer cell lines, which proliferated independent of estrogen addition (Fig. 5a and b, N-E2(+) versus N-E2(−), black solid versus black dashed lines). We then compared proliferation under normoxic versus hypoxic conditions. Interestingly, the ER-α negative cell lines, MDA-MB-231 and MDA-MB-453, which are claudin low and luminal B molecular subtypes respectively, grew identically at 20% or 1% O_2_ (Fig. 5a and b; Black (normoxic) versus gray (hypoxic) lines). In contrast, proliferation of ER-α positive cells was strongly decreased by low oxygen levels despite the addition of estradiol to the media (Fig. 5c-f; N-E2(+) versus N-E2(−), black solid versus dashed lines). ER-α positive cell growth was indistinguishable whether cells were grown in estrogen-free media under normoxic conditions or estrogen-containing media under hypoxic conditions. To further understand the reduced growth rates in hypoxic conditions, cell cycle analysis at day 3–6 of hypoxic versus normoxic culture was performed. We found that all samples underwent cell cycle arrest: three of our four exemplar cell lines stall in G1 (BT474, T47D, ZR75B) in hypoxia, as indicated by an increase in the percent of cells in G1 versus S and G2/M phases of the cell cycle (Fig. 5g). The fourth cell line, MCF7, exhibits S-phase stalling, as demonstrated by the expanded percent of cells in S-phase and smaller G1 and G2/M fractions. In further experiments we found no change in Annexin V reactivity at day 3 of normoxic versus hypoxic culture, suggesting little apoptotic induction (data not shown). These data reveal a strong relationship between ER-α activity and cell proliferation that is modulated by hypoxia in a variety of tumor genetic backgrounds.

## Discussion

The recent move towards precision medicine, which aims to prospectively identify clinical drug responders versus non-responders, requires a parallel preclinical move to the experimental use of panels of tumor-derived cell lines to more accurately assess possible therapeutic responses and frequencies. Here, we bridge that gap to define the effects of hypoxia on ER-α expression and activity in a panel of breast cancer cell lines that represent common genomic variations seen in human ER-α positive tumors. Previous work with exemplar cell lines, primarily MCF7 or ZR75, has suggested a role for hypoxia in reducing ER-α expression [28, 29, 31, 44]. However the literature taken as a whole contains multiple, conflicting results particularly with regard to mechanisms and with no consideration given to the potential influence of genetic background variations that commonly occur in ER-α positive tumors. Thus, common responses that might be relevant for improving hormonal therapies remain unclear.

We surprisingly find that reduced oxygen availability produces rapid reduction of ER-α protein levels in all of our genetically-diverse cell lines (Fig. 1a, b). This is largely effected by enhanced proteolysis, although a theoretical role for altered ER-α -specific translation attenuation cannot be entirely ruled out (Fig. 3d, Additional file 3D). Hypoxia reduces ER-α levels in essentially all of the cells within the population (Fig. 1c). We also uniquely find that the mechanisms that enhance proteolysis of ER-α during hypoxia can be activated even in breast tumor derivatives that are naturally ER-α negative and very distantly related, for example the claudin low molecular phenotype represented by MDA-MB-231 (Fig. 3b). Thus, we propose that the molecular mechanisms that promote ER-α proteolysis are likely to be fundamental to the hypoxic response, and that ER-α may be one of a potentially large suite of proteins that undergo proteolytic regulation during hypoxic adaptation. In support of this hypothesis, others have shown hypoxia-induced proteolysis of the α-secretases ADAM10 and TACE without alteration in mRNA levels in neuroblastoma [45], and of the MYC oncogene in human colon carcinoma cells and primary human keratinocytes, involving the ubiquitin ligases FBXW7 and DDB1, and cathepsins D and S [46]. Similarly, the ubiquitin ligase Siah2 has been implicated in hypoxic proteolysis of the HIF-1α prolyl hydroxylases PHD1 and PHD3 [47] and of the E1 subunit of α-ketogluterate dehydrogenase complex [48].

There is significant controversy regarding hypoxia-induced alterations in *ESR1* mRNA levels. We find that none of the 10 cell lines in our panel show an obvious reduction in *ESR1* mRNA abundance after 24 h of hypoxic culture, although they exhibit significant, rapid reduction in the level of ER-α protein (Fig. 1a versus Fig. 3a). This concurs with the findings in MCF7 [28] and ZR75 [31] which assessed mRNA at a similar time point (24 h). In other reports, a reduction in the level of *ESR1* mRNA involving ERK kinase activity [29] were determined after 72 h of hypoxic culture, which may explain their discrepant results. Finally, reports of early (8–24 h), HIF-1α-dependent decreases in the levels of *ESR1* mRNA [30] in MCF7 and T47D, contrast directly with our findings. These studies differed in that the level of *ESR1* mRNA was analyzed in estrogen-starved cells in the presence versus absence of hypoxia. We predict that our results are most likely to represent the acute, common behavior of *bona fide* breast tumors undergoing hypoxic adaptation in an estrogenic environment such as the breast or metastatic site in the female body.

Using shRNAs targeting *HIF1A* and *HIF2A* we directly demonstrate that HIF-1α, but not HIF-2α commonly decreases ER-α protein levels (Fig. 2c versus Additional file 7B) in hypoxic culture conditions. However, these shRNA studies only decreased the levels of HIF-1α mRNA and protein without entirely eliminating expression, which may explain why ER-α protein levels were not more robustly restored in hypoxia in these cell lines (Fig. 2c). Similarly, transient overexpression of a stable HIF-1α in normoxic conditions did not entirely eliminate ER-α expression (Fig. 2d), which also might be explained as a technical failure to transfect all cells in the population. Alternatively, other hypoxia-induced factors that function independently of HIF-1α expression may also influence ER-α stability. For example, the weak HIF-2α expression seen under normoxic conditions might in fact be stabilizing ER-α in an unknown fashion, and the reduction in both molecules might be somehow linked in hypoxia. Proteases activated by hypoxia in other systems including cathepsins B, D, and S [46, 49] and calpains [50–52] as well as inducible E3 ubiquitin ligases such as SIAH2 [48, 53] may also provide HIF-1α - independent ER-α degradation in hypoxia. However it is clear from our studies that HIF-1α plays an important role in ER-α regulation.

Since mRNA levels of *ESR1* do not decrease in response to hypoxia in any of the cell lines we examined (Fig. 3a), the decreased protein levels cannot result from direct transcriptional repression by HIF heterodimers or of HIF-induced transcriptional repressors. Thus HIF activity must indirectly influence factors that enhance proteolysis of ER-α. This may simply involve transcriptional induction of ubiquitin ligases or proteases, or more indirectly by induction of a known HIF-dependent transcriptional target such as miRNA-155 [54, 55] or a methyltransferase such as WDR5 [56]. Alternatively, HIF-1α and ER-α might compete for transcriptional co-activators, for example P300/CBP (reviewed in [2]). In this scenario, increased hypoxic levels of HIF-1α could result in ER-α degradation, due to the lack of co-activators that prevent proteasome targeting. Finally, ER-α may compete with HIF-1α for VHL-mediated ubiquitination and proteasome targeting, as reported in renal cell carcinomas [57]. Others have implicated HIF-1α in ER-α regulation by overexpression of chimeric HIF-1α-VP16 [44] and by HIF-1α silencing by siRNA in a single cell line, MCF7 [30]. However, our results demonstrate that this activity is more common among breast tumors than the results in MCF7 would suggest, and reveal that HIF-1α inhibitors should be further explored in preclinical studies as co-therapeutics in the endocrine therapy setting.

Finally, we find that the hypoxia-induced reduction in ER-α levels decreases ER-α -directed transcription (Fig. 4a-d) and significantly reduces proliferation (Fig. 5c-f) without inducing overt cell death in each cell line tested. Unexpectedly, this lack of estrogen signaling is not overcome by switching to other proliferative signaling pathways in any of our cells over the time courses we assessed (Fig. 5, 6–16 days). This leads us to speculate that chronic residence in hypoxic environments may be one explanation for the later disease reoccurrence seen in some ER-α positive breast cancer patients. We hypothesize that a dormant, non-proliferating phenotype may allow tumor cells to persist undetected, until conditions change to oxygenate the environment or until cells eventually switch to proliferation via other signaling pathways. We note that hypoxia can induce protein phosphatase 2A, which preserves viability without proliferation in glioblastoma multiforme-derived cells [58] and we speculate that a similar mechanism may be at play in our system. Furthers studies involving chronic hypoxia are ongoing in our laboratory. The hypoxia sensitivity of ER-α positive, but not ER-α negative, basal-like cell lines reported here may also provide one explanation for clinical observations that ER-α positive tumors grow more slowly than ER-α negative tumors, providing patients with better 5 year survival statistics. We speculate that ER-α negative tumors will grow in vivo regardless of environmental oxygen levels, whereas growth of ER-α positive tumors will be limited to that proportion of the tumor that is oxygenated. Finally, accounting for differential expression of ER-α and other hypoxia sensitive molecules in hypoxic regions of tumors offers one explanation for the intra-tumor heterogeneity of ER-α expression seen in many clinical breast tumor sections.

Several publications have shown that ER-α levels are reduced in regions of potentially hypoxic tissue in human clinical specimens, although in each case the authors were unable to determine whether this resulted from a lack of nutrients, oxygen, or an increase in cellular waste products. For example, Cooper et al. [28] examined cells adjacent to necrotic breast tumor cores by immuno-histochemical methods, and found that ER-α protein levels were decreased, HIF-1α levels increased, and expression of the HIF-1α target gene CA-IX was increased. Similarly, Kronblad et al. [29] also demonstrated reduced ER-α and increased HIF-1α halos of tumor cells surrounding necrotic cores. Finally Lloyd, et al. [59] measured the spatial distribution of ER-α reactivity in relationship to vascularity in breast tissue sections by immuno-histochemical staining. Each study found reduced ER-α levels either adjacent to necrotic cores or distal from vasculature. Unlike clinical results, we can definitely say that hypoxia alone is enough to produce reduced ERα protein levels in ER-α positive tumor cells despite variation in other accompanying genetic mutations.

## Conclusions

This study is the first comprehensive overview of the effects of low environmental oxygen on ER-α abundance and transcriptional activity in a diverse set of culture adapted tumor cell lines independently derived from ER-α positive breast cancer patients. Our uniform, detailed analysis of this panel reveals surprising and potentially actionable homogeneity in the effect of hypoxia on ER-α. Our findings that there is a common requirement for the proteasome and HIF-1α in ER-α repression in hypoxic environments, among many different breast cancer genomes implies that ubiquitin ligase or proteasome inhibitors might be widely used in the clinic to potentiate the effects of hormonal therapies or delay/prevent therapeutic resistance. Similarly, the common requirement for HIF-1α activity implies that specific HIF inhibitors currently under development may also and perhaps more specifically improve current endocrine therapies. Thus this analysis reveals multiple new ideas for badly-needed improvements in endocrine-based therapeutics.

## Additional files

Additional file 1: Cell line characteristics. Data assembled from the citations listed in the table. nd, not determined; wt, wildtype; hom, homozygous; het, heterozygous; mis, missense; bal, balanced. Numbers from Neve 2006 represent relative signal intensity on Western blot. (DOCX 20 kb)
Additional file 2: Standard culture media has estrogenic effects. ER activity analyzed by a reporter assay at normoxia in estradiol-free media, E_2_ (−); standard culture RPMI or DMEM; or defined estrogen media, E_2_ (+). (A) MCF7 cell lines and (B) T47D cell lines. See cell culture methods section for media compositions. (PDF 362 kb)
Additional file 3: (A) qPCR analysis of mRNA levels of *ESR1* and *HIF1A* at normoxia (N) or hypoxia (H) (1% O_2_, 24 h) treated with DMSO or MG132. Relative mRNA levels normalized to *TBP*. (B-D) Averages and standard deviations of band intensities calculated for all repeats of each western blot in Fig. 3. Specific band intensities normalized to the loading control bands (β-actin). (B) HIF-1α and ER-α protein from Fig. 3b. (C) phospho-p70-S6K and phospho-4E-BP1 protein from Fig. 3c. (D) HIF-1α and ER-α protein from Fig. 3d. The ER-α graphs represent ER-α normalized to compare the increase in ER-α protein levels generated by MG132 treatment in normoxic versus hypoxic conditions. * MCF7 ER-α levels are significantly different, alpha = 0.05, *p* = 0.044). (PDF 1787 kb)
Additional file 4: Averages of ER-α band intensities for all repeats of each western blot in Fig. 1. (A) from all experimental replicates of Fig. 1a. (B) for Fig. 1b. (C) for Fig. 1d. Specific band intensities normalized to the loading control bands (β-actin). (PDF 551 kb)
Additional file 5: Averages and standard deviations of ER-α band intensities calculated for all repeats of each western blot in Fig. 1a. Specific band intensities normalized to the loading control bands (β-actin). Calculations derived from at least three independent experiments. (DOCX 15 kb)
Additional file 6: Averages and standard deviations of band intensities calculated for all repeats of each western blot in Fig. 1b. Specific band intensities normalized to the loading control bands (β-actin). Calculations derived from at least three independent experiments. (DOCX 15 kb)
Additional file 7: (A) qPCR analysis of mRNA levels for *HIF2A* in MCF7, T47D and ZR75B transfected with shScramble (shScr) or sh*HIF2A*. Relative mRNA levels normalized to *TBP*. (Change in *HIF2A* levels: **p* = 0.005 MCF7, **p* = 0.019 T47D, **p* = 0.012 ZR75B). (B) Representative western blots of HIF-2α, ER-α and β-actin protein from MCF7, T47D and ZR75B with either shScramble (shScr) or h*HIF2A* at normoxia and hypoxia (1% O_2_, 24 h). β-actin is used as a loading control. (C-F) Averages and standard deviations of band intensities calculated for all repeats of each western blot in Fig. 2. Specific band intensities normalized to the loading control bands (β-actin). (C) HIF-1α and HIF-2α protein from Fig. 2a. (D) HIF-1α and ER-α protein from Fig. 2c. (E) HIF-1α and ER-α protein from Fig. 2d. (F) HIF-1α and ER-α protein from Additional file 7B. (PDF 1953 kb)
Additional file 8: Averages and standard deviations of band intensities calculated for all repeats of each western blot in Fig. 2a. Specific band intensities normalized to the loading control bands (β-actin). Calculations derived from at least three independent experiments. (DOCX 17 kb)
Additional file 9: Averages and standard deviations of band intensities calculated for all repeats of each western blot in Fig. 2c. Specific band intensities normalized to the loading control bands (β-actin). Calculations derived from at least three independent experiments. (DOCX 17 kb)
Additional file 10: Averages and standard deviations of band intensities calculated for all repeats of each western blot in Additional file 7B. Specific band intensities normalized to the loading control bands (β-actin). Calculations derived from at least three independent experiments. (DOCX 16 kb)
Additional file 11: Averages and standard deviations of band intensities calculated for all repeats of each western blot in Fig. 2d. Specific band intensities normalized to the loading control bands (β-actin). Calculations derived from at least three independent experiments. (DOCX 15 kb)
Additional file 12: Averages and standard deviations of band intensities calculated for all repeats of each western blot in Fig. 3c. Specific band intensities normalized to the loading control bands (β-actin). Calculations derived from at least three independent experiments. (DOCX 15 kb)
Additional file 13: Normalized western blot values for ER-α protein levels from blots used to generate Fig. 3d, and the graphs in Additional file 3D. These values were used to test the relative increase in ER-α levels induced by MG132 treatment in normoxic versus hypoxic conditions for statistical significance. (DOCX 15 kb)
Additional file 14: Averages and standard deviations of band intensities calculated for all repeats of each western blot in Fig. 3d for HIF-1 alpha. Specific band intensities normalized to the loading control bands (β-actin). Calculations derived from at least three independent experiments. (DOCX 15 kb)
Additional file 15: Averages and standard deviations of band intensities calculated for all repeats of each western blot in Fig. 3b. Specific band intensities normalized to the loading control bands (β-actin). Calculations derived from at least three independent experiments. (DOCX 15 kb)

## Abbreviations

ADAM10: A disintegrin and metalloproteinase domain-containing protein 10
ARNT: Aryl hydrocarbon receptor nuclear translocator
CBP: Coactivator binding protein
CMV: Cytomegalovirus
DDB1: DNA damage-binding protein 1
DMEM: Dulbecco’s modified Eagle’s media
ECL: Enhanced chemiluminescence
ERE: Estrogen Response Element
ERK: Extracellular signal-regulated kinase
ER-α: Estrogen receptor alpha
*ESR1*: Estrogen receptor 1 gene
FBS: Fetal bovine serum
FBXW7: F-box and WD repeat domain containing 7
HER2: Human epidermal growth factor receptor 2
HIF: Hypoxia induced factor
HIF-1α: Hypoxia induced factor 1 alpha
HIF-1β: Hypoxia induced factor 1 beta
HRE: Hypoxic-response elements
MYC: Avian myelocytomatosis viral oncogene homolog
O_2_: Oxygen
PBS: Phosphate buffered saline
PHD: Prolyl-4-hydroxylase
PI3KCA: Phosphoinositide-3-kinase alpha catalytic subunit
PI3KCA: Phosphatidylinositol-4, 5-bisphosphate 3-kinase catalytic subunit alpha isoform
Rb: Retinoblastoma
RPMI: Roswell Park Memorial Institute media
shRNA: Short hairpin ribonucleic acid
SIAH2: Seven in absentia homolog 2
TACE: A disintegrin and metalloproteinase domain-containing protein 17
TP53: Tumor protein 53
VHL: Von Hippel–Lindau
VP16: Herpes simplex virus, viral protein 16
WDR5: WD repeat domain 5
