# Supplementary material for: Genome-independent hypoxic repression of estrogen receptor alpha in breast cancer cells
Source: BMC Cancer. 2017 Mar 20;17:203. doi: 10.1186/s12885-017-3140-9 (PMC5358051; doi:10.1186/s12885-017-3140-9)
Supplement: Additional file 1: — Cell line characteristics. Data assembled from the citations listed in the table. nd, not determined; wt, wildtype; hom, homozygous; het, heterozygous; mis, missense; bal, balanced. Numbers from Neve 2006 represent relative signal intensity on Western blot. (DOCX 20 kb) [file 12885_2017_3140_MOESM1_ESM.docx]

| NAME | **MCF7** | **T47D** | **ZR-75-B** | **BT474** | **CAMA-1** | **MDA-MB-453** | **HCC1428** | **MDA-MB-175-VII** | **600MPE** | **MDA-MB-361** | **M-MDA-MB-231** | **LY2** |
| --- | --- | --- | --- | --- | --- | --- | --- | --- | --- | --- | --- | --- |
| Neve 2006 [34]; Western blot quantitation | | | | |  |  |  |  |  |  |  |  |
| AKT1-P | 0 | 0 | 472 | 2487 | 2329 | 1521 | nd | 3197 | 0 | 670 | 0 | 733 |
| ERBB2 | 512 | 2092 | 3642 | 6882 | 1127 | 3057 | nd | 4497 | 1342 | 7478 | 1991 | 1854 |
| ERBB2-P | 0 | 0 | 77861 | 21847 | 0 | 6927 | nd | 0 | 0 | 1402 | 0 | 0 |
| ESR1 | 11778 | 7303 | 8623 | 3917 | 3726 | 1141 | nd | 458 | 6937 | 7147 | 542 | 10892 |
| IGF1R | 20750 | 11827 | 1433 | 5091 | 202 | 1539 | nd | 2659 | 2452 | 9300 | 7859 | 3295 |
| PTEN | 15183 | 17796 | 4 | 24360 | 1790 | 111397 | nd | 10596 | 9270 | 9970 | 18564 | 4072 |
| TP53 | 0 | 27032 | 262 | 10699 | 17118 | 0 | nd | 0 | 0 | 0 | 36742 | 196 |
| COSMIC [8]; sequence analysis | | | | |  |  |  |  |  |  |  |  |
| PIKC3A | het E545K | het H104R | nd | het K111N | wt | het H1047R | wt | wt | nd | E545K, K567R | wt | nd |
| PTEN | wt | wt | nd | wt | het mis | het E307K | wt | wt | nd | wt | wt | nd |
| p53 | wt | hom mis | nd | hom mis | hom mis | het mis | wt | wt | nd | hom non | het mis | nd |
| Stemke-Hale 2008 [6]; sequence analysis | | | | | | | | | | | | |
| PIKC3A | E545K | H1047R | wt | K111N | wt | H1047R | nd | nd | wt | E545K | wt | E545K |
| PTEN | wt | wt | mut | wt | mut | wt | nd | nd | wt | wt | wt | wt |

| NAME | **MCF7** | **T47D** | **ZR-75-B** | **BT474** | **CAMA-1** | **MDA-MB-453** | **HCC1428** | **MDA-MB-175-VII** | **600MPE** | **MDA-MB-361** | **M-MDA-MB-231** | **LY2** |
| --- | --- | --- | --- | --- | --- | --- | --- | --- | --- | --- | --- | --- |
| Hollenstalle 2010 [7]; copy number: | | | | | | | | | | | | |
| CDH1 mut and meth | wt | wt | nd | wt | c.1712-1G>A hom | c.1913G>A hom | nd | wt | c.1138-21_1138del22 hom | wt | methylated | nd |
| CCND1 amplification | bal/+ | bal/+ | nd | gain/+ | gain/++ | bal/+ | nd | gain/+ | gain/++ | bal/+ | bal/+ | nd |
| ERBB2 amplification | bal/- | bal/- | nd | gain/++ | bal/+ | bal/++ | nd | bal/- | bal/+ | gain/++ | bal/- | nd |
| p16 mut and meth | c.1_471del471 hom | methylated | nd | wt | wt | wt | nd | wt | methylated | c.156G>C hom | c.1_471del471 hom | nd |
| p53 mutation | wt | c.580C>T hom | nd | c.853G>A hom | c.839G>C hom | c.991_1182del192 hom | nd | wt | wt | c.166G>T hom | c.839G>A hom | nd |
| PIK3CA mutation | c.1633G>A het | c.3140A>G het | nd | c.333G>C het | wt | c.3140A>G het | nd | wt | wt | c.1633G>A (hetero); c.1700A>G het | wt | nd |
| PTEN mutation | wt | wt | nd | wt | c.274G>C; c.802_803insTAGG/834_837delCTTC compound het | c.919G>A het | nd | wt | wt | wt | wt | nd |

Additional file 1: Cell line characteristics. Data assembled from the citations listed in the table. nd, not determined; wt, wildtype; hom, hompozygous; het, heterozygous; mis, missense; bal, balanced. Numbers from Neve 2006 [34] represent relative signal intensity on Western blot.
