# Supplementary figures and images for: Genome-independent hypoxic repression of estrogen receptor alpha in breast cancer cells
Source: BMC Cancer. 2017 Mar 20;17:203. doi: 10.1186/s12885-017-3140-9 (PMC5358051; doi:10.1186/s12885-017-3140-9)

**A****MCF7**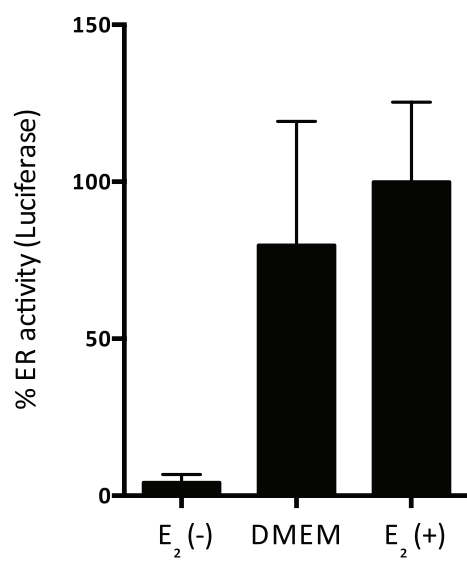**B****T47D**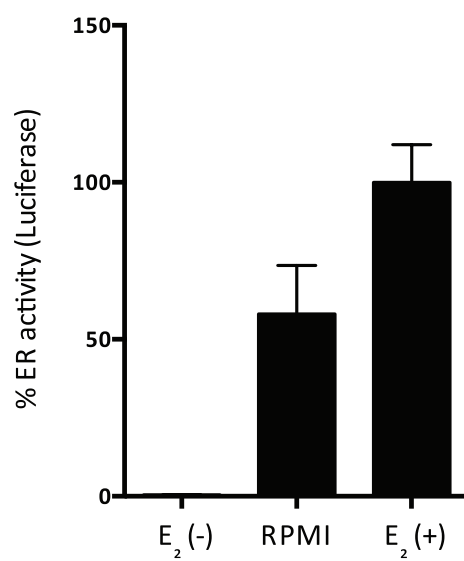

Supplement: Additional file 2: — Standard culture media has estrogenic effects. ER activity analyzed by a reporter assay at normoxia in estradiol-free media, E2 (−); standard culture RPMI or DMEM; or defined estrogen media, E2 (+). (A) MCF7 cell lines and (B) T47D cell lines. See cell culture methods section for media compositions. (PDF 362 kb) [file 12885_2017_3140_MOESM2_ESM.pdf]

A

*ESR1*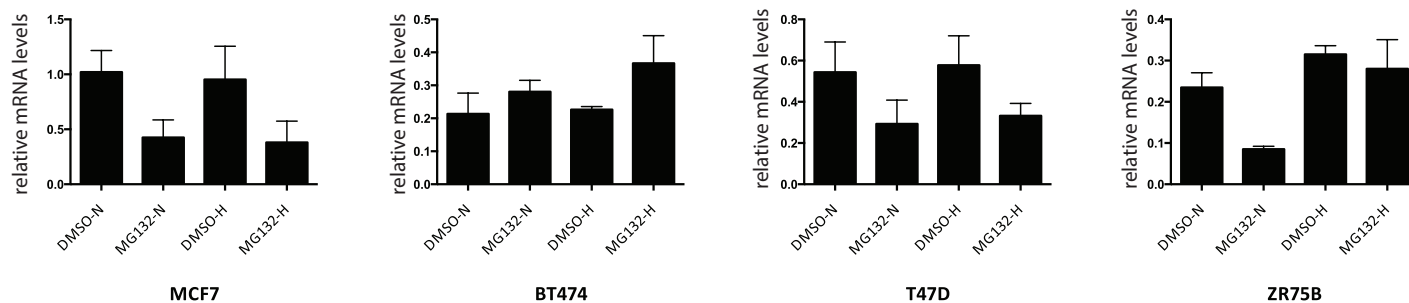*HIF1A*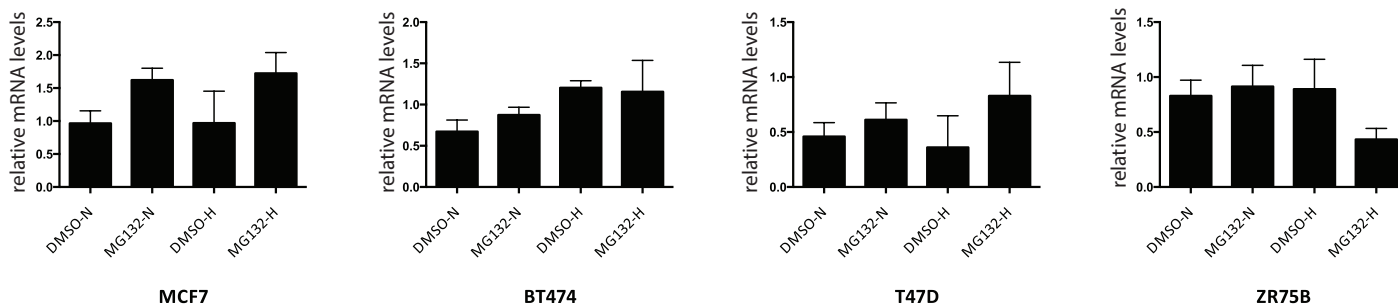

B

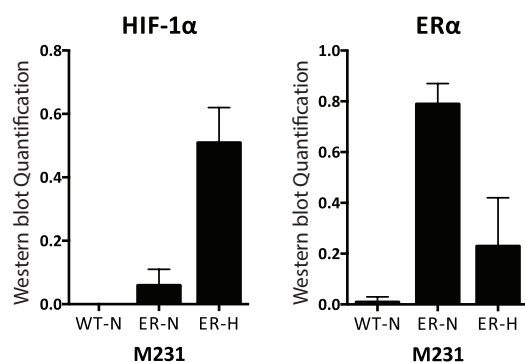

C

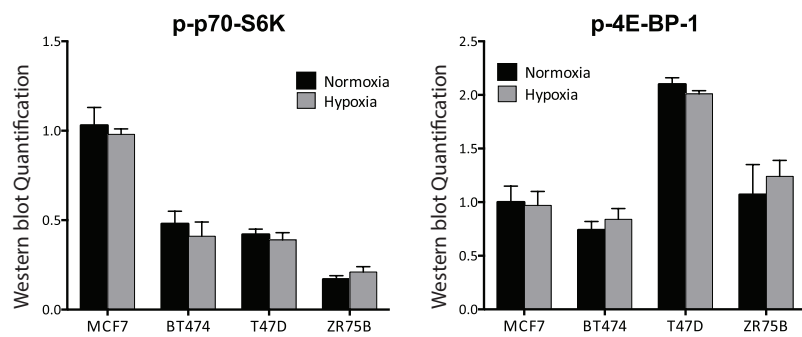

D

*HIF1-α*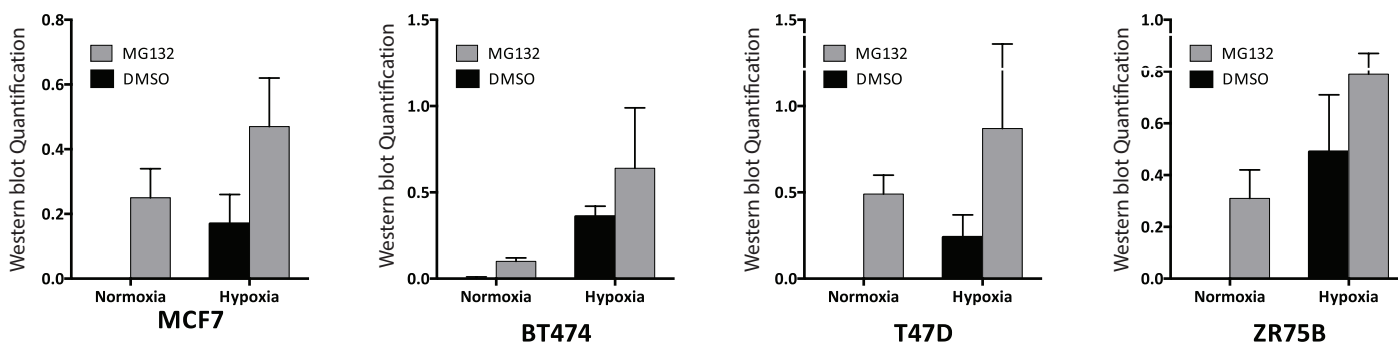*ER-α*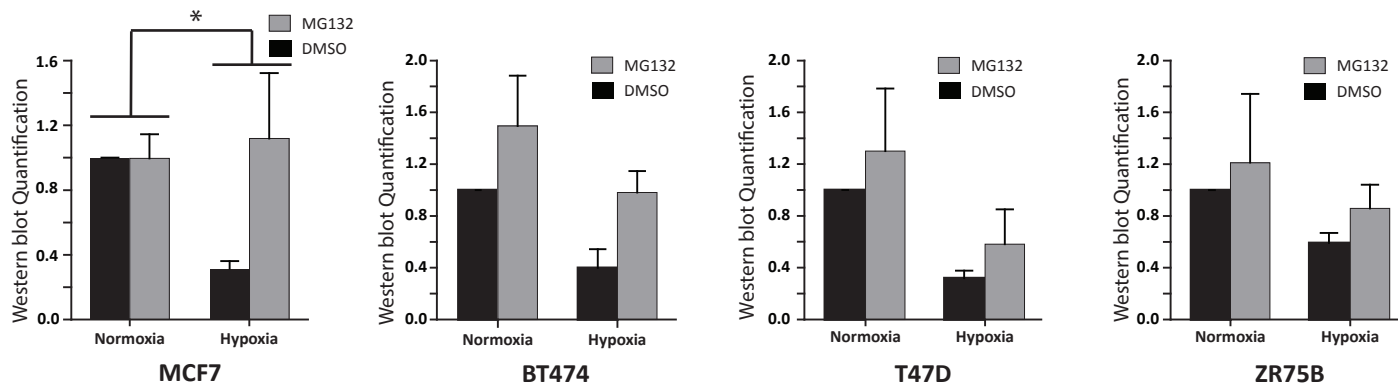

Supplement: Additional file 3: — (A) qPCR analysis of mRNA levels of ESR1 and HIF1A at normoxia (N) or hypoxia (H) (1% O2, 24 h) treated with DMSO or MG132. Relative mRNA levels normalized to TBP. (B-D) Averages and standard deviations of band intensities calculated for all repeats of each western blot in Fig. 3. Specific band intensities normalized to the loading control bands (β-actin). (B) HIF-1α and ER-α protein from Fig. 3b. (C) phospho-p70-S6K and phospho-4E-BP1 protein from Fig. 3c. (D) HIF-1α and ER-α protein from Fig. 3d. The ER-α graphs represent ER-α normalized to compare the increase in ER-α protein levels generated by MG132 treatment in normoxic versus hypoxic conditions. * MCF7 ER-α levels are significantly different, alpha = 0.05, p = 0.044). (PDF 1787 kb) [file 12885_2017_3140_MOESM3_ESM.pdf]

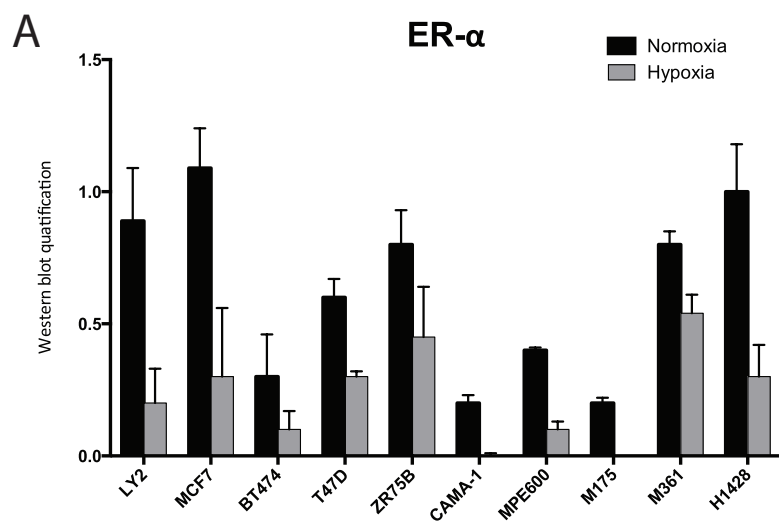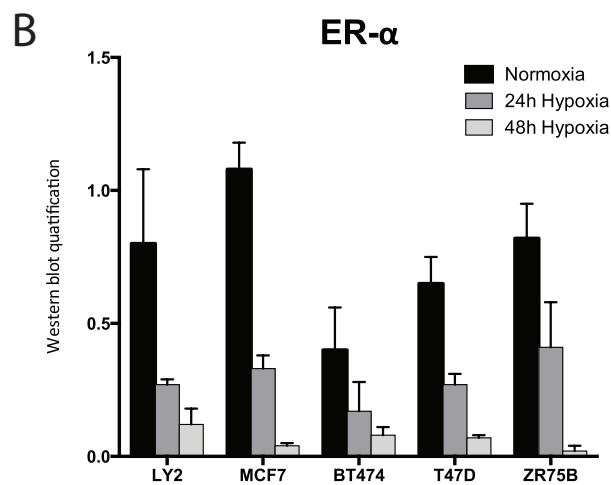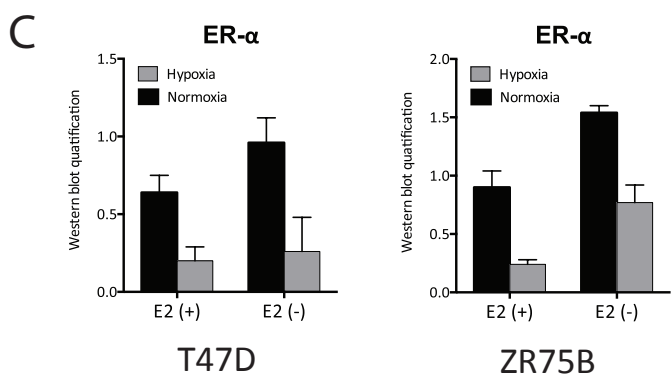

Supplement: Additional file 4: — Averages of ER-α band intensities for all repeats of each western blot in Fig. 1. (A) from all experimental replicates of Fig. 1a. (B) for Fig. 1b. (C) for Fig. 1d. Specific band intensities normalized to the loading control bands (β-actin). (PDF 551 kb) [file 12885_2017_3140_MOESM4_ESM.pdf]

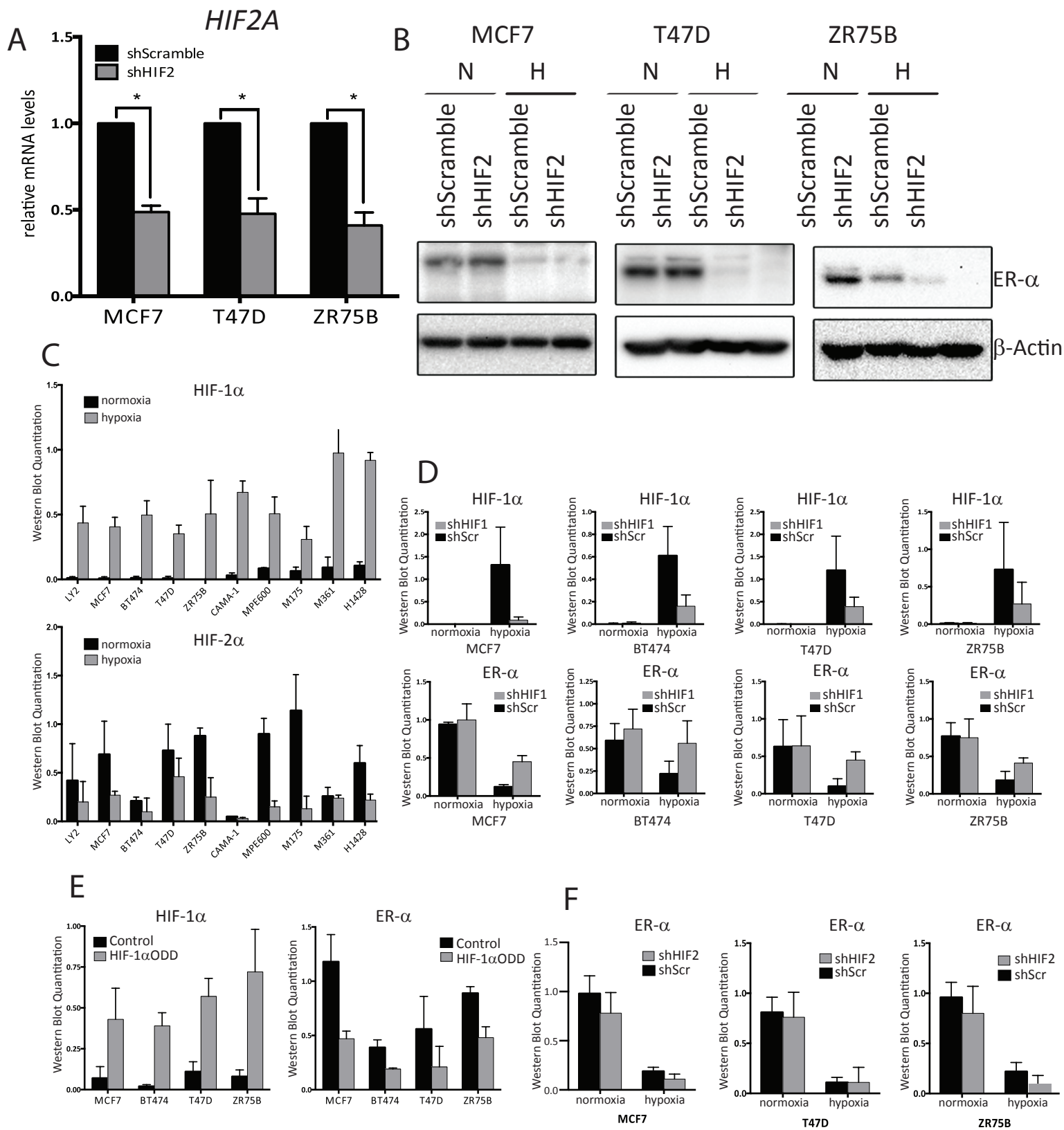

Supplement: Additional file 7: — (A) qPCR analysis of mRNA levels for HIF2A in MCF7, T47D and ZR75B transfected with shScramble (shScr) or shHIF2A. Relative mRNA levels normalized to TBP. (Change in HIF2A levels: *p = 0.005 MCF7, *p = 0.019 T47D, *p = 0.012 ZR75B). (B) Representative western blots of HIF-2α, ER-α and β-actin protein from MCF7, T47D and ZR75B with either shScramble (shScr) or hHIF2A at normoxia and hypoxia (1% O2, 24 h). β-actin is used as a loading control. (C-F) Averages and standard deviations of band intensities calculated for all repeats of each western blot in Fig. 2. Specific band intensities normalized to the loading control bands (β-actin). (C) HIF-1α and HIF-2α protein from Fig. 2a. (D) HIF-1α and ER-α protein from Fig. 2c. (E) HIF-1α and ER-α protein from Fig. 2d. (F) HIF-1α and ER-α protein from Additional file 7B. (PDF 1953 kb) [file 12885_2017_3140_MOESM7_ESM.pdf]
