# Supplementary material for: Genome-independent hypoxic repression of estrogen receptor alpha in breast cancer cells
Source: BMC Cancer. 2017 Mar 20;17:203. doi: 10.1186/s12885-017-3140-9 (PMC5358051; doi:10.1186/s12885-017-3140-9)
Supplement: Additional file 5: — Averages and standard deviations of ER-α band intensities calculated for all repeats of each western blot in Fig. 1a. Specific band intensities normalized to the loading control bands (β-actin). Calculations derived from at least three independent experiments. (DOCX 15 kb) [file 12885_2017_3140_MOESM5_ESM.docx]

|  | **Normoxia** | | **Hypoxia** | |
| --- | --- | --- | --- | --- |
|  | Mean | St.Dev | Mean | St.Dev |
| LY2 | 0.89 | 0.20 | 0.20 | 0.13 |
| MCF7 | 1.09 | 0.15 | 0.30 | 0.26 |
| BT474 | 0.30 | 0.16 | 0.10 | 0.07 |
| T47D | 0.60 | 0.07 | 0.30 | 0.02 |
| ZR75B | 0.80 | 0.13 | 0.45 | 0.19 |
| CAMA-1 | 0.20 | 0.03 | 0.00 | 0.01 |
| MPE600 | 0.40 | 0.01 | 0.10 | 0.03 |
| M175 | 0.20 | 0.02 | 0.00 | 0.00 |
| M361 | 0.80 | 0.05 | 0.54 | 0.07 |
| H1428 | 1.00 | 0.18 | 0.30 | 0.12 |

**Additional File 5.**Western blot quantifications of ERα protein from figure 1a. Protein intensity was normalized to the loading control (β-actin). Mean and standard deviation of at least three independent experiments.
