# Supplementary material for: Genome-independent hypoxic repression of estrogen receptor alpha in breast cancer cells
Source: BMC Cancer. 2017 Mar 20;17:203. doi: 10.1186/s12885-017-3140-9 (PMC5358051; doi:10.1186/s12885-017-3140-9)
Supplement: Additional file 6: — Averages and standard deviations of band intensities calculated for all repeats of each western blot in Fig. 1b. Specific band intensities normalized to the loading control bands (β-actin). Calculations derived from at least three independent experiments. (DOCX 15 kb) [file 12885_2017_3140_MOESM6_ESM.docx]

|  | Normoxia | | 24h Hypoxia | | 48h Hypoxia | |
| --- | --- | --- | --- | --- | --- | --- |
|  | Mean | St.Dev | Mean | St.Dev | Mean | St.Dev |
| LY2 | 0.8 | 0.28 | 0.27 | 0.02 | 0.12 | 0.06 |
| MCF7 | 1.08 | 0.1 | 0.33 | 0.05 | 0.04 | 0.01 |
| BT474 | 0.4 | 0.16 | 0.17 | 0.11 | 0.08 | 0.03 |
| T47D | 0.65 | 0.1 | 0.27 | 0.04 | 0.07 | 0.01 |
| ZR75B | 0.82 | 0.13 | 0.41 | 0.17 | 0.02 | 0.02 |

**Additional File 6.** Western blot quantifications of ERα protein from figure 1b. Protein intensity was normalized to the loading control (β-actin). Mean and standard deviation of at least three independent experiments.
