# Supplementary material for: Genome-independent hypoxic repression of estrogen receptor alpha in breast cancer cells
Source: BMC Cancer. 2017 Mar 20;17:203. doi: 10.1186/s12885-017-3140-9 (PMC5358051; doi:10.1186/s12885-017-3140-9)
Supplement: Additional file 8: — Averages and standard deviations of band intensities calculated for all repeats of each western blot in Fig. 2a. Specific band intensities normalized to the loading control bands (β-actin). Calculations derived from at least three independent experiments. (DOCX 17 kb) [file 12885_2017_3140_MOESM8_ESM.docx]

|  | HIF-1α | | | | HIF-2α | | | |
| --- | --- | --- | --- | --- | --- | --- | --- | --- |
|  | Normoxia | | Hypoxia | | Normoxia | | Hypoxia | |
|  | Mean | St.Dev | Mean | St.Dev | Mean | St.Dev | Mean | St.Dev |
| LY2 | 0.01 | 0.01 | 0.44 | 0.13 | 0.42 | 0.38 | 0.20 | 0.21 |
| MCF7 | 0.01 | 0.01 | 0.41 | 0.07 | 0.69 | 0.34 | 0.27 | 0.04 |
| BT474 | 0.01 | 0.01 | 0.50 | 0.11 | 0.21 | 0.04 | 0.10 | 0.14 |
| T47D | 0.01 | 0.02 | 0.35 | 0.07 | 0.73 | 0.27 | 0.46 | 0.19 |
| ZR75B | 0.00 | 0.00 | 0.51 | 0.26 | 0.88 | 0.08 | 0.25 | 0.20 |
| CAMA-1 | 0.03 | 0.02 | 0.67 | 0.09 | 0.05 | 0.00 | 0.03 | 0.01 |
| MPE600 | 0.09 | 0.01 | 0.51 | 0.13 | 0.90 | 0.16 | 0.15 | 0.06 |
| M175 | 0.06 | 0.03 | 0.31 | 0.10 | 1.14 | 0.37 | 0.13 | 0.13 |
| M361 | 0.09 | 0.08 | 0.98 | 0.26 | 0.26 | 0.09 | 0.24 | 0.03 |
| H1428 | 0.11 | 0.03 | 0.92 | 0.06 | 0.60 | 0.18 | 0.22 | 0.06 |

**Additional File 8.** Western blot quantifications of HIF-1α and HIF-2α protein from figure 2a. Protein intensity was normalized to the loading control (β-actin). Mean and standard deviation of at least three independent experiments.
