# Supplementary material for: Genome-independent hypoxic repression of estrogen receptor alpha in breast cancer cells
Source: BMC Cancer. 2017 Mar 20;17:203. doi: 10.1186/s12885-017-3140-9 (PMC5358051; doi:10.1186/s12885-017-3140-9)
Supplement: Additional file 9: — Averages and standard deviations of band intensities calculated for all repeats of each western blot in Fig. 2c. Specific band intensities normalized to the loading control bands (β-actin). Calculations derived from at least three independent experiments. (DOCX 17 kb) [file 12885_2017_3140_MOESM9_ESM.docx]

|  |  | HIF-1α | | | | ER-α | | | |
| --- | --- | --- | --- | --- | --- | --- | --- | --- | --- |
|  |  | shScramble | | shHIF1 | | shScramble | | shHIF1 | |
|  |  | Mean | St.Dev | Mean | St.Dev | Mean | St.Dev | Mean | St.Dev |
| MCF7 | Normoxia | 0.00 | 0.00 | 0.00 | 0.00 | 0.94 | 0.03 | 1.00 | 0.21 |
|  | Hypoxia | 1.32 | 0.84 | 0.09 | 0.07 | 0.12 | 0.03 | 0.45 | 0.08 |
| BT474 | Normoxia | 0.00 | 0.01 | 0.01 | 0.01 | 0.59 | 0.19 | 0.72 | 0.22 |
|  | Hypoxia | 0.61 | 0.26 | 0.16 | 0.10 | 0.22 | 0.14 | 0.56 | 0.25 |
| T47D | Normoxia | 0.00 | 0.01 | 0.00 | 0.00 | 0.63 | 0.36 | 0.64 | 0.40 |
|  | Hypoxia | 1.20 | 0.76 | 0.39 | 0.21 | 0.10 | 0.10 | 0.45 | 0.11 |
| ZR75B | Normoxia | 0.01 | 0.01 | 0.01 | 0.01 | 0.77 | 0.18 | 0.75 | 0.25 |
|  | Hypoxia | 0.73 | 0.63 | 0.27 | 0.29 | 0.18 | 0.12 | 0.41 | 0.07 |

**Additional File 9.** Western blot quantifications of HIF-1α and ER-α protein from figure 2c. Protein intensity was normalized to the loading control (β-actin). Mean and standard deviation of at least three independent experiments.
