# Supplementary material for: Genome-independent hypoxic repression of estrogen receptor alpha in breast cancer cells
Source: BMC Cancer. 2017 Mar 20;17:203. doi: 10.1186/s12885-017-3140-9 (PMC5358051; doi:10.1186/s12885-017-3140-9)
Supplement: Additional file 10: — Averages and standard deviations of band intensities calculated for all repeats of each western blot in Additional file 7B. Specific band intensities normalized to the loading control bands (β-actin). Calculations derived from at least three independent experiments. (DOCX 16 kb) [file 12885_2017_3140_MOESM10_ESM.docx]

|  |  | ER-α | | | |
| --- | --- | --- | --- | --- | --- |
|  |  | shScrammble | | shHIF2 | |
|  |  | Mean | St.Dev | Mean | St.Dev |
| MCF7 | Normoxia | 0.98 | 0.18 | 0.78 | 0.21 |
|  | Hypoxia | 0.19 | 0.04 | 0.11 | 0.05 |
| T47D | Normoxia | 0.81 | 0.15 | 0.76 | 0.25 |
|  | Hypoxia | 0.11 | 0.05 | 0.11 | 0.15 |
| ZR75B | Normoxia | 0.96 | 0.15 | 0.80 | 0.27 |
|  | Hypoxia | 0.22 | 0.09 | 0.08 | 0.10 |

**Additional File 10.** Western blot quantifications of ER-α protein from Additional file 7B. Protein intensity was normalized to the loading control (β-actin). Mean and standard deviation of at least three independent experiments.
