# Supplementary material for: Genome-independent hypoxic repression of estrogen receptor alpha in breast cancer cells
Source: BMC Cancer. 2017 Mar 20;17:203. doi: 10.1186/s12885-017-3140-9 (PMC5358051; doi:10.1186/s12885-017-3140-9)
Supplement: Additional file 11: — Averages and standard deviations of band intensities calculated for all repeats of each western blot in Fig. 2d. Specific band intensities normalized to the loading control bands (β-actin). Calculations derived from at least three independent experiments. (DOCX 15 kb) [file 12885_2017_3140_MOESM11_ESM.docx]

|  | HIF-1α | | | | ER-α | | | |
| --- | --- | --- | --- | --- | --- | --- | --- | --- |
|  | Control | | HIF-1aODD | | Control | | HIF-1aODD | |
|  | Mean | St.Dev | Mean | St.Dev | Mean | St.Dev | Mean | St.Dev |
| MCF7 | 0.07 | 0.07 | 0.43 | 0.19 | 1.18 | 0.25 | 0.47 | 0.07 |
| BT474 | 0.02 | 0.01 | 0.39 | 0.08 | 0.39 | 0.07 | 0.19 | 0.01 |
| T47D | 0.11 | 0.06 | 0.57 | 0.11 | 0.56 | 0.3 | 0.21 | 0.19 |
| ZR75B | 0.08 | 0.04 | 0.72 | 0.26 | 0.89 | 0.06 | 0.48 | 0.1 |

**Additional File 11.** Western blot quantifications of HIF-1α and ER-α protein from figure 2d. Protein intensity was normalized to the loading control (β-actin). Mean and standard deviation of at least three independent experiments.
