# Supplementary material for: Genome-independent hypoxic repression of estrogen receptor alpha in breast cancer cells
Source: BMC Cancer. 2017 Mar 20;17:203. doi: 10.1186/s12885-017-3140-9 (PMC5358051; doi:10.1186/s12885-017-3140-9)
Supplement: Additional file 12: — Averages and standard deviations of band intensities calculated for all repeats of each western blot in Fig. 3c. Specific band intensities normalized to the loading control bands (β-actin). Calculations derived from at least three independent experiments. (DOCX 15 kb) [file 12885_2017_3140_MOESM12_ESM.docx]

|  | p-p70-S6K | | | | p-4E-BP1 | | | |
| --- | --- | --- | --- | --- | --- | --- | --- | --- |
|  | Normoxia | | Hypoxia | | Normoxia | | Hypoxia | |
|  | Mean | St.Dev | Mean | St.Dev | Mean | St.Dev | Mean | St.Dev |
| MCF7 | 1.03 | 0.10 | 0.98 | 0.03 | 1.00 | 0.15 | 0.97 | 0.13 |
| BT474 | 0.48 | 0.07 | 0.41 | 0.08 | 0.74 | 0.08 | 0.84 | 0.10 |
| T47D | 0.42 | 0.03 | 0.39 | 0.04 | 2.10 | 0.06 | 2.01 | 0.03 |
| ZR75B | 0.17 | 0.02 | 0.21 | 0.03 | 1.07 | 0.28 | 1.24 | 0.15 |

**Additional File 12.** Western blot quantifications of phosphor-p70-S6K and phosphor-4E-BP1 protein from figure 3c. Protein intensity was normalized to the loading control (β-actin). Mean and standard deviation of at least three independent experiments.
