# Supplementary material for: Genome-independent hypoxic repression of estrogen receptor alpha in breast cancer cells
Source: BMC Cancer. 2017 Mar 20;17:203. doi: 10.1186/s12885-017-3140-9 (PMC5358051; doi:10.1186/s12885-017-3140-9)
Supplement: Additional file 13: — Normalized western blot values for ER-α protein levels from blots used to generate Fig. 3d, and the graphs in Additional file 3D. These values were used to test the relative increase in ER-α levels induced by MG132 treatment in normoxic versus hypoxic conditions for statistical significance. (DOCX 15 kb) [file 12885_2017_3140_MOESM13_ESM.docx]

|  |  | ER alpha | | | |
| --- | --- | --- | --- | --- | --- |
|  |  | DMSO | | MG132 | |
|  |  | Mean | St. Dev | Mean | St. Dev |
| MCF7 | Normoxia | 1.00 | 0.00 | 1.00 | 0.15 |
|  | Hypoxia | 0.32 | 0.05 | 1.12 | 0.4 |
| BT474 | Normoxia | 1.00 | 0.00 | 1.5 | 0.39 |
|  | Hypoxia | 0.4 | 0.14 | 0.98 | 0.17 |
| T47D | Normoxia | 1.00 | 0.00 | 1.30 | 0.48 |
|  | Hypoxia | 0.32 | 0.06 | 0.58 | 0.27 |
| ZR-75-B | Normoxia | 1.00 | 0.00 | 1.34 | 0.37 |
|  | Hypoxia | 0.73 | 0.32 | 1.17 | 0.36 |

**Additional File 13.** Normalized western blot values for ER-alpha protein levels from blots used to generate Figure 3d, and the graphs in Additional file 3D. These values were used to test the relative increase in ER-alpha levels induced by MG132 treatment in normoxic versus hypoxic conditions.
