# Supplementary material for: Genome-independent hypoxic repression of estrogen receptor alpha in breast cancer cells
Source: BMC Cancer. 2017 Mar 20;17:203. doi: 10.1186/s12885-017-3140-9 (PMC5358051; doi:10.1186/s12885-017-3140-9)
Supplement: Additional file 14: — Averages and standard deviations of band intensities calculated for all repeats of each western blot in Fig. 3d for HIF-1 alpha. Specific band intensities normalized to the loading control bands (β-actin). Calculations derived from at least three independent experiments. (DOCX 15 kb) [file 12885_2017_3140_MOESM14_ESM.docx]

|  |  | HIF-1 alpha | | | |
| --- | --- | --- | --- | --- | --- |
|  |  | DMSO | | MG132 | |
|  |  | Mean | St. Dev | Mean | St. Dev |
| MCF7 | Normoxia | 0.00 | 0.00 | 0.25 | 0.09 |
|  | Hypoxia | 0.17 | 0.09 | 0.47 | 0.15 |
| BT474 | Normoxia | 0.00 | 0.01 | 0.10 | 0.02 |
|  | Hypoxia | 0.36 | 0.06 | 0.64 | 0.35 |
| T47D | Normoxia | 0.00 | 0.00 | 0.49 | 0.11 |
|  | Hypoxia | 0.24 | 0.13 | 0.87 | 0.49 |
| ZR-75-B | Normoxia | 0.00 | 0.00 | 0.31 | 0.11 |
|  | Hypoxia | 0.49 | 0.22 | 0.79 | 0.08 |

**Additional File 14.** Western Blot quantifications of HIF-1 alpha from blots for figure 3d. Protein intensity was normalized to the loading control (beta actin). Mean and standard deviation of at least three independent experiments.
