# Supplementary material for: Genome-independent hypoxic repression of estrogen receptor alpha in breast cancer cells
Source: BMC Cancer. 2017 Mar 20;17:203. doi: 10.1186/s12885-017-3140-9 (PMC5358051; doi:10.1186/s12885-017-3140-9)
Supplement: Additional file 15: — Averages and standard deviations of band intensities calculated for all repeats of each western blot in Fig. 3b. Specific band intensities normalized to the loading control bands (β-actin). Calculations derived from at least three independent experiments. (DOCX 15 kb) [file 12885_2017_3140_MOESM15_ESM.docx]

|  | HIF-1α | | ER-α | |
| --- | --- | --- | --- | --- |
|  | Mean | St.Dev | Mean | St.Dev |
| WT-N | 0.00 | 0.00 | 0.01 | 0.02 |
| ER-N | 0.06 | 0.05 | 0.79 | 0.08 |
| ER-H | 0.51 | 0.11 | 0.23 | 0.19 |

**Additional File 15.** Western blot quantifications of HIF-1α and ER-α protein from figure 3b. Protein intensity was normalized to the loading control (β-actin). Mean and standard deviation of at least three independent experiments.
